# Supplementary material for: Efficacy of Combination Chemotherapy Using a Novel Oral Chemotherapeutic Agent, FTD/TPI, with Ramucirumab Murine Version DC101 in a Mouse Syngeneic Cancer Transplantation Model
Source: J Clin Med. 2020 Dec 15;9(12):4050. doi: 10.3390/jcm9124050 (PMC7765280; doi:10.3390/jcm9124050)
Supplement: Supplementary file 1 [file jcm-09-04050-s001.pdf]

Journal of
Clinical Medicine

MDPI

Supplymental Figures, Tables and Schemes

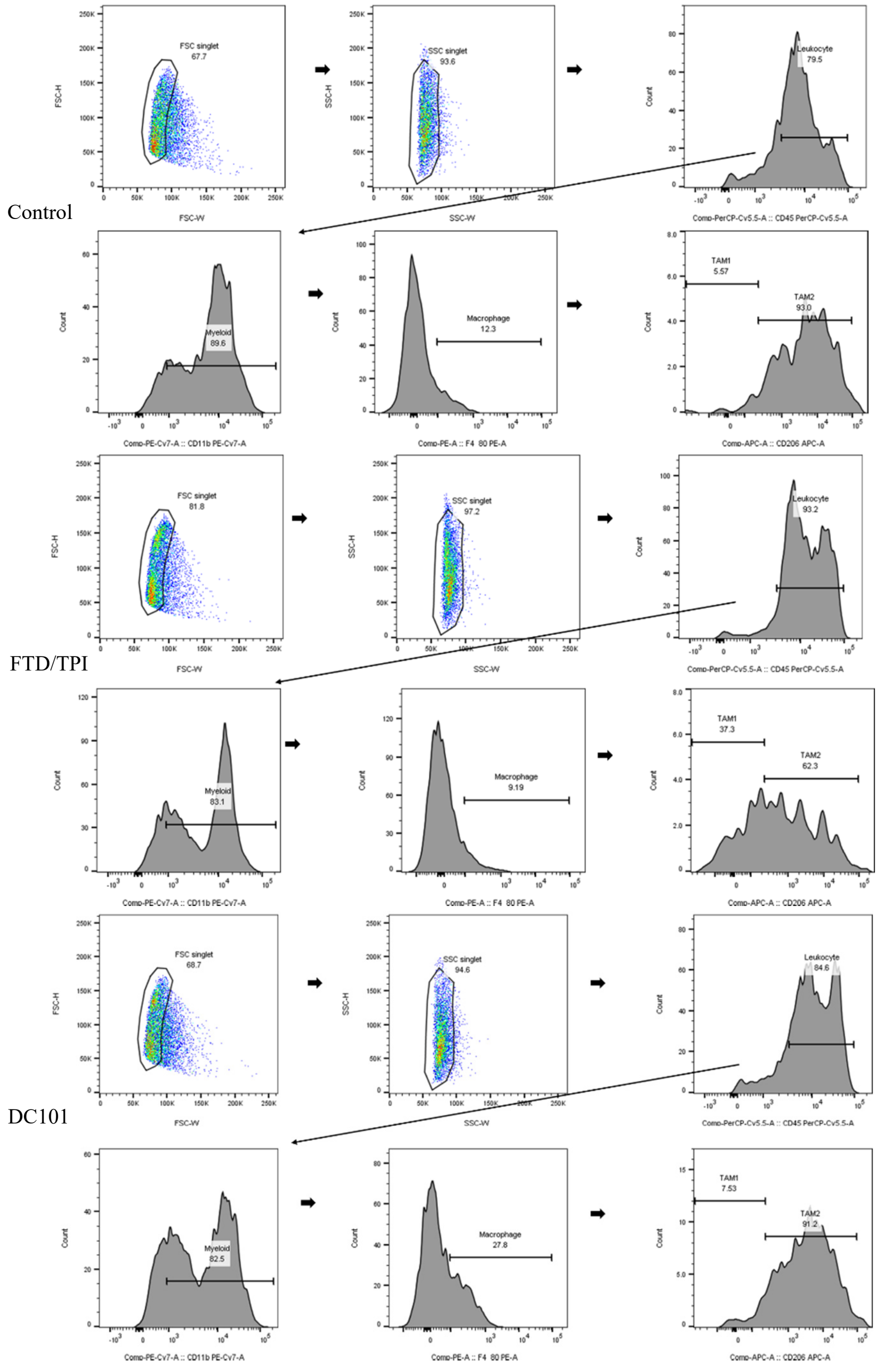

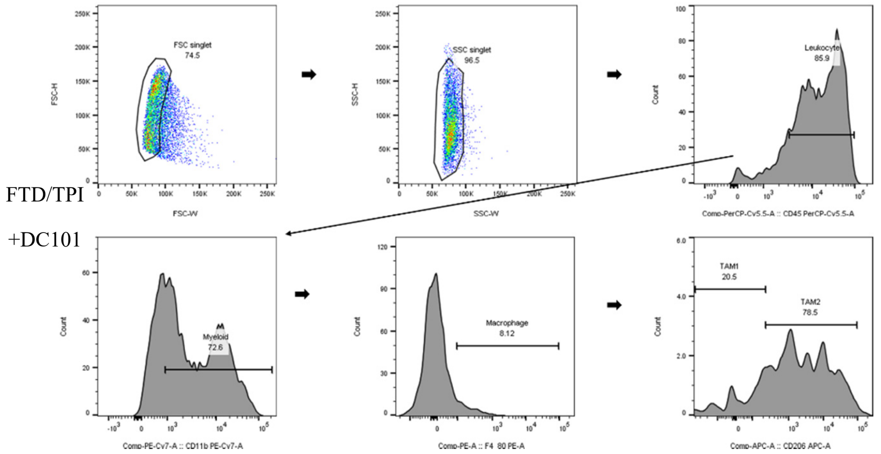

Supplemental Figure 1. The representative image of TAM population selection graph plot.

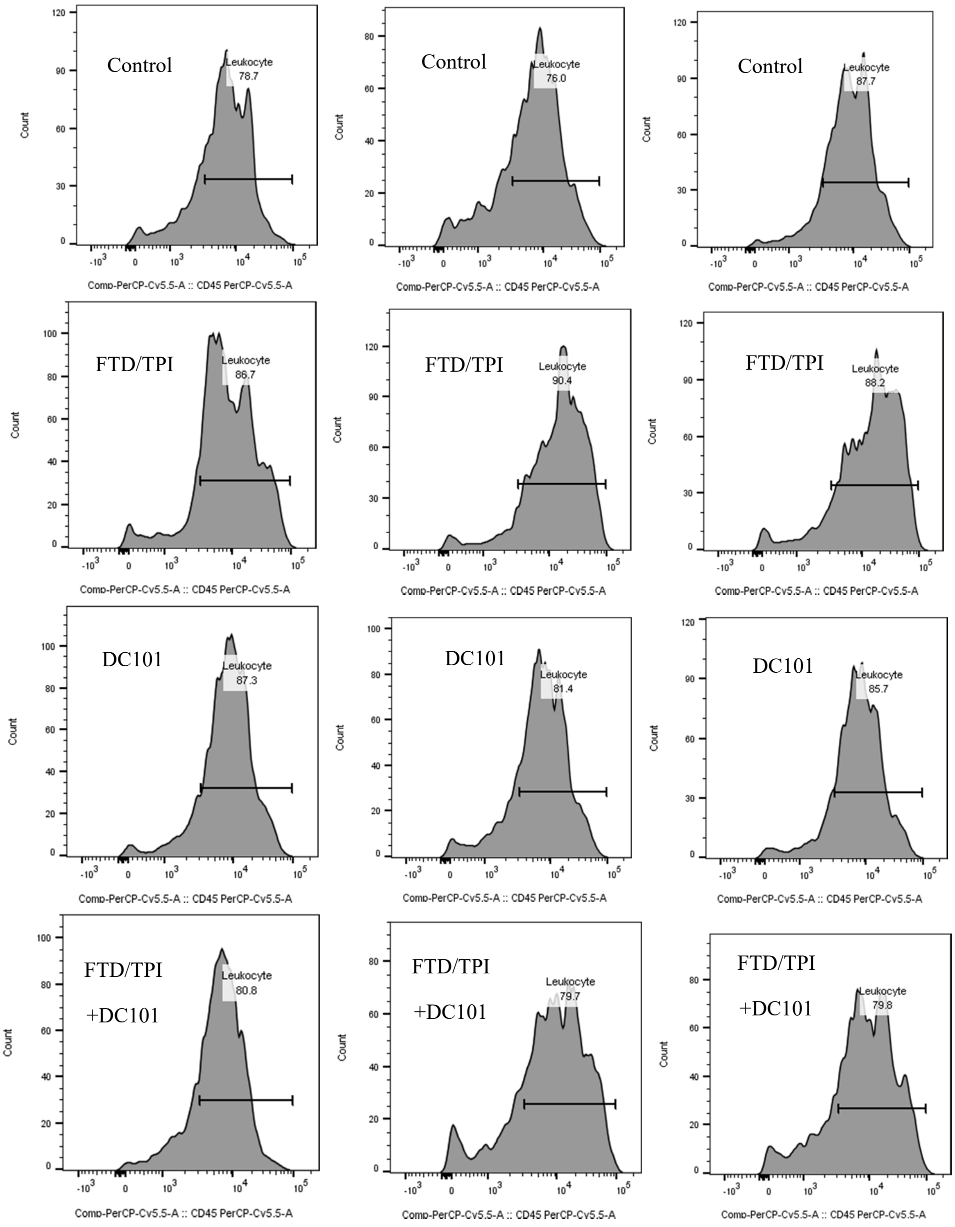

Supplemental Figure 2. The proportion of each TIL CD45 positive population depicted in Figure 4. c.

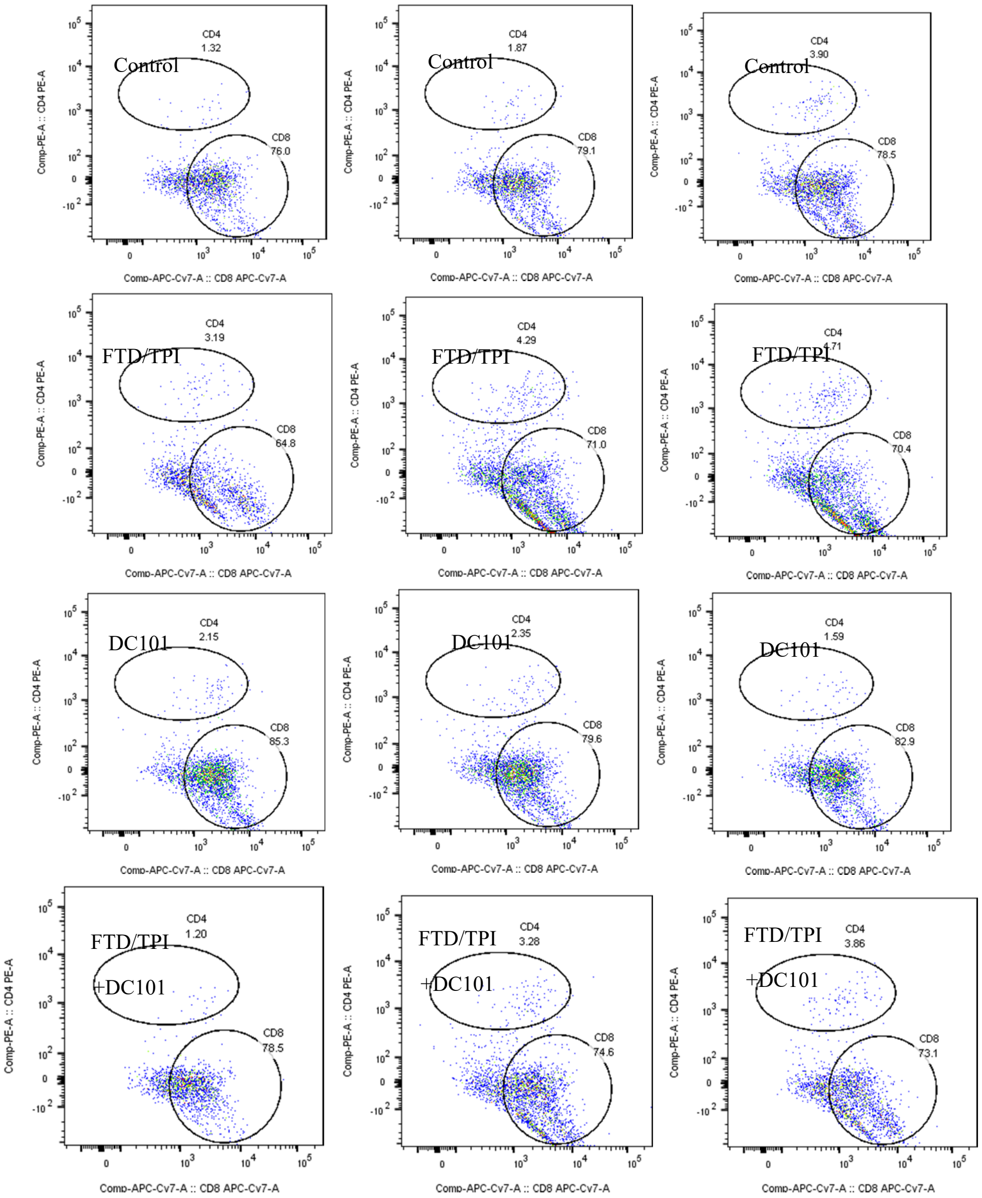

Supplemental Figure 3. CD4+/CD8+ population quadrant plot depicted.in the Figure. 4 c.

Supplemental Table 1. Body weight changes in mice implanted with murine colorectal tumor CT26 after treatment with FTD/TPI and DC101.

Drug	Schedule Route	Number of Animals	BWC[a)] (%, mean ± SE)	p-value Aspin-Welch's		
				Control	FTD/TPI	DC101
Control	Days 1-5, 8-12 (sid, po) + Days 1, 3, 5, 7, 9, 11, and 13 (sid, ip)	6	15.8 ± 2.0			
FTD/TPI 200 mg/kg-bwt/day	Days 1-5 and 8-12 (sid, po)	6	7.3 ± 2.2	0.016		
DC101 0.8 mg into each mice	Days 1, 3, 5, 7, 9, 11, and 13 (sid, ip)	6	8.0 ± 2.0	0.021		
FTD/TPI 200 mg/kg-bwt/day +DC101 0.8 mg into each mice	Days 1-5 and 8-12 (sid, po) + Days 1, 3, 5, 7, 9, 11, and 13(sid, ip)	6	0.1 ± 1.3	<0.001	0.018	0.008

(a): Body weight change (BWC, %; mean±SD) on Day 15 were calculated according to the following formula: BWC (%) = [(BW on Day 15) − (BW on Day 0)] / (BW on Day 0) × 100. SE: standard error,: p-value by Aspin-Welch t test as compared to the control and monotherapy group BWC, body weight change.

© 2020 by the authors. Submitted for possible open access publication under the terms and conditions of the Creative Commons Attribution (CC BY) license (http://creativecommons.org/licenses/by/4.0/).
